# Gonadectomy and Its Association with Orthopedic and Neoplastic Disorders: A Retrospective Study in Belgium—Part I (Bitches)

**DOI:** 10.3390/life16050707

**Published:** 2026-04-22

**Authors:** Guillaume Domain, Florin Petrisor Posastiuc, Joke Lannoo, Lotte Spanoghe, Jeroen Dewulf, Ann Van Soom

**Affiliations:** 1 Department of Internal Medicine, Reproduction and Population Medicine, Faculty of Veterinary Medicine, Ghent University, 9820 Merelbeke, Belgium; florin.posastiuc@ugent.be (F.P.P.); joke.lannoo@ugent.be (J.L.); lotte.spanoghe@ugent.be (L.S.); jeroen.dewulf@ugent.be (J.D.); ann.vansoom@ugent.be (A.V.S.); 2 Department of Clinical Sciences II, Faculty of Veterinary Medicine, University of Agronomic Sciences and Veterinary Medicine, 050097 Bucharest, Romania

**Keywords:** gonadectomy, spaying, bitches, orthopedic disorders, neoplasia, retrospective study, risk factors

## Abstract

Gonadectomy is performed in bitches to prevent unwanted reproduction and reduce the risk of sex hormone-related conditions. However, growing evidence suggests that the timing of spaying may influence long-term susceptibility to non-reproductive diseases. This retrospective case–control study (2013–2023) evaluated the association between timing of spaying and the development of orthopedic and neoplastic disorders in a Belgian referral-hospital population. Cases included bitches diagnosed with cranial cruciate ligament rupture, hip dysplasia, elbow dysplasia, lymphoma, mast cell tumor, osteosarcoma, or hemangiosarcoma, while disease-free bitches served as controls. Associations between disease occurrence and spaying status were assessed using multivariable logistic regression adjusted for age, weight category, and body condition score. Age at gonadectomy (<12 vs. ≥12 months) and timing relative to the first estrus were evaluated in separate models. Spaying <12 months of age was associated with increased odds of all conditions compared with intact females. Spaying ≥12 months of age was associated with lower odds of several orthopedic and neoplastic outcomes compared with early spaying, although odds were not always comparable to those in intact females. Large body size and higher body condition score were independently associated with increased odds of orthopedic outcomes. These findings support individualized spaying strategies rather than a universal age threshold.

## 1. Introduction

Gonadectomy is one of the most frequently performed surgical procedures in small animal practice. In females, it is widely recommended to prevent unwanted pregnancy and reduce the risk of mammary tumors, pyometra, and other reproductive disorders [1,2,3,4]. However, the proportion of spayed bitches varies markedly across regions. While neutering rates exceed 70% in North America, they remain considerably lower in most European countries [5,6,7,8,9]. In recent years, attention has increasingly turned to whether gonadectomy, and particularly the age at which it is performed, may have long-term effects on health and longevity [10,11,12].

Traditionally, concerns related to spaying in bitches were limited to urinary incontinence, changes in coat quality or behavior, and weight gain [2,13,14]. Over the last decade, however, several studies have reported associations between (prepubertal) gonadectomy and an increased risk of orthopedic disorders and certain neoplastic diseases, although these associations appear to vary according to tumor type and study population [9,15,16,17]. Although causality has not yet been established, these findings raise important biological questions about how early hormonal deprivation may influence physiological systems beyond the reproductive tract [4,14,18]. These observations align with the Developmental Origins of Health and Disease (DOHaD) concept, which emphasizes that physiological systems are particularly sensitive during early developmental stages [19,20]. Disruptive events during this period, such as abrupt withdrawal of ovarian hormones, may affect growth plate closure, immune maturation, and tissue differentiation, thereby potentially modifying disease susceptibility later in life [19].

Despite rising scientific interest, most available data originate from North America, where spaying is more common and typically performed at a younger age than in Europe. Large-scale European studies focusing specifically on female dogs remain scarce, limiting the ability to make regionally adapted recommendations. Moreover, few studies have simultaneously assessed orthopedic and neoplastic disorders in a multi-breed population. Therefore, the aim of this retrospective case–control study was to evaluate the association between gonadectomy, its timing, and the risk of specific orthopedic disorders (cranial cruciate ligament rupture [CCLR], hip dysplasia [HD], elbow dysplasia [ED]) and neoplastic diseases (lymphoma [LSA], mast cell tumor [MCT], hemangiosarcoma [HSA], osteosarcoma [OSA]) in bitches in a Belgian referral-hospital population. We hypothesized that early-life deprivation of ovarian hormones would be associated with higher odds of the disorders examined.

## 2. Materials and Methods

### 2.1. Patients and Inclusion Criteria

Medical records of all bitches presented at the Veterinary Medical Teaching Hospital of Ghent University between 1 January 2013 and 31 December 2023 were retrospectively reviewed. The hospital uses proprietary software (Claris FileMaker Pro version 22.0.1.68) to generate a standardized electronic medical record for each patient, which includes owner demographic information, signalment of the animal, anamneses, physical examination findings, laboratory test results, diagnoses and treatments.

Screening of medical records was performed using disease-related keywords selected to capture common abbreviations, Dutch and English terminology, and word stems for the predefined orthopedic and neoplastic conditions, with an asterisk (*) applied as a wildcard character to expand the search scope. Retrieved records were subsequently reviewed for eligibility, and bitches were included only if a definitive diagnosis of one of the conditions of interest (CCLR, HD, ED, LSA, MCT, OSA, and HSA) was documented. Bitches with a suspected but unconfirmed diagnosis were excluded from the case and control group.

Bitches with orthopedic disorders were typically presented for lameness, reduced mobility, or joint pain. A definitive diagnosis was established by a board-certified orthopedist or a resident under their supervision based on orthopedic examination findings and radiographic confirmation. Since Ghent University is one of the official screening centers for HD and ED in purebred breeding dogs in Belgium, bitches younger than 18 months were excluded from HD and ED analyses in both the case and control groups to avoid overrepresentation of young, asymptomatic, intact bitches. Cases were identified using the search terms “vkb”, “ccr”, “cruc*”, “kruis*”, “hd”, “heupdysplasie”, “heup*”, “hipdysp*”, “hip*”, “ocd”, “lpc”, “lpa”, “mcd”, “elleboogdysplasie”, “fmcp”, “incongruen”, “dissecan*”, and “coron*”.

Neoplastic disorders were diagnosed by a board-certified internist or a resident under their supervision, based on the presence of a mass or enlarged lymph nodes, results from blood examinations, diagnostic imaging, histopathology, and/or cytology. Cases were identified using the search terms “lympho*”, “lymfo*”, “has”, “sarc*”, “osteo*”, “bone”, “mct”, “mastcel”, and “mast*”.

The control group consisted of clinically healthy bitches or bitches presented for medical issues unrelated to the orthopedic or neoplastic disorders investigated in this study. These visits included vaccination, reproductive management, or blood donation, but also otitis, nail injuries, intoxication, dental procedures, ocular problems, wound treatment, or fracture management, provided that none of the investigated disorders had been previously documented in the medical record. Control cases were identified using the search terms “castr*”, “tand*”, “oog*”, “vaccin*”, “bloed*”, “sperm*”, “gezond”, “otitis”, “nagel”, “fractuur”, “wond*”, “intox*”, and “hbc”.

### 2.2. Data Collection

Data extracted from the medical records included breed, age at symptom onset (or at diagnosis if symptom onset was not specified), body condition score (BCS), neuter status, age at gonadectomy and whether gonadectomy occurred before or after the first estrus. Age at symptom onset was preferred when available because, in a referral-hospital population, diagnosis may occur after a variable delay from the first clinically recognized signs. When symptom onset was not clearly documented, age at diagnosis was used as the most objective available reference point. For each bitch, the recorded BCS corresponded to the clinical visit at which the diagnosis of the condition of interest was established, or, in control animals, the visit at which inclusion criteria were met. Neutered bitches were further classified as having undergone early (<12 months of age) or late (≥12 months of age) gonadectomy. The 12-month threshold was chosen as a clinically relevant cut-off, already described in the literature to distinguish early gonadectomy from later procedures, and to allow the use of a single threshold across a study population including bitches of different breed sizes [15,21,22].

Breeds were categorized into three weight classes according to the breed’s standard adult weight: small (≤10 kg), medium (10–25 kg), and large (≥25 kg) [23]. In mixed-breed bitches, weight class was assigned based on recorded body weight, taking BCS into account. Body condition score was determined using a 9-point scale widely applied in canine clinical practice, which estimates body fat through visual inspection and palpation [24]. Scores range from 1 (emaciated) to 9 (obese), with 4 and 5 considered ideal. This method was chosen over body weight because it accounts for differences in individual morphology, providing a more reliable assessment of overweight status [25].

Bitches with missing data for a specific variable were excluded only from analyses involving that variable, but were retained for analyses of other variables for which complete data were available.

### 2.3. Statistical Analysis

The normality of continuous variables was assessed using the Shapiro–Wilk test, with a significance threshold set at α = 0.05. Descriptive statistics are presented as medians with interquartile ranges (IQR), including the 25th and 75th percentiles. Group comparisons were performed using Kruskal–Wallis and Mann–Whitney U tests.

Univariable logistic regression analyses were initially performed to evaluate potential associations between candidate risk factors (age, BCS, and weight class) and each outcome of interest. Variables demonstrating a potential association (*p* < 0.20) in the univariable models were retained for inclusion in the corresponding multivariable analyses to avoid confounding. Separate multivariable logistic regression models were constructed for each disease outcome. For each outcome, the retained covariates were included together with the neutering variable of interest. Because age at gonadectomy and timing relative to the first estrus are closely related exposure measures and may be collinear, they were evaluated in separate multivariable models rather than entered simultaneously. Statistical significance was defined as *p* < 0.05, and effect sizes were expressed as odds ratios (OR) with 95% confidence intervals (95% CI). All statistical analyses were performed using SPSS Statistics version 26.0 (IBM Corp., Armonk, NY, USA).

## 3. Results

### 3.1. Study Population

A total of 2655 bitches were included in the study period, of which 1066 (40.2%) were intact and 1589 (59.8%) were spayed. The median age was 72 months (IQR: 38–107 months), and the median BCS was 5 (IQR: 4–6). Timing of spaying was available for 1073/1589 spayed females: 541 (50.4%) were early-spayed (<12 months) and 532 (49.6%) were late-spayed (≥12 months). Information on the first estrus was available for 1040/1589 spayed females, with 333 (32.0%) spayed before and 707 (68.0%) after their first estrus.

The distribution of BCS differed between intact and spayed females (*p* < 0.001). Although the median BCS was identical in both groups, spayed females showed a right-shifted distribution, with a higher 90th percentile (8 vs. 7).

Table 1 summarizes the descriptive characteristics of each outcome group stratified by neuter status.

### 3.2. Orthopedic Disorders

#### 3.2.1. Cranial Cruciate Ligament Rupture

Body size and BCS were significantly associated with CCLR. Bitches ≥25 kg had more than twice the odds of CCLR compared with those ≤10 kg (OR = 2.35, 95% CI 1.81–3.06; *p* < 0.001), and more than three times the odds compared with those weighing 10–25 kg (OR = 3.31, 95% CI 2.51–4.36; *p* < 0.001). Bitches weighing 10–25 kg also had higher odds compared with bitches ≤10 kg (OR = 1.41, 95% CI 1.01–1.96; *p* < 0.05). Each one-point increase in BCS increased the odds by 33% (OR = 1.33, 95% CI 1.23–1.45; *p* < 0.001), whereas age was not significantly associated with CCLR.

Neuter status was significantly associated with CCLR. Spayed females had higher odds than intact bitches (OR = 2.45, 95% CI 1.98–3.03; *p* < 0.001). Early-spayed bitches had the highest risk, with 4.09 times the odds of intact females (95% CI 2.97–5.63; *p* < 0.001) and 2.56 times the odds of late-spayed females (95% CI 1.77–3.72; *p* < 0.001). Late-spayed bitches also had moderately increased odds compared with intact females (OR = 1.59, 95% CI 1.15–2.21; *p* < 0.01).

**Figure 1:**
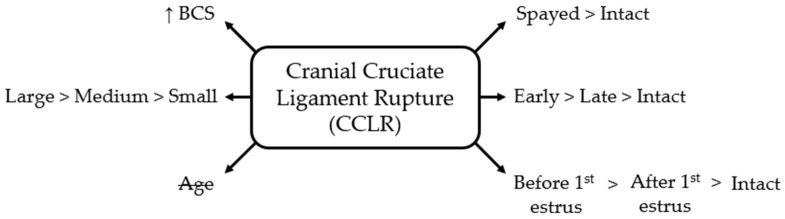
Schematic overview of factors associated with cranial cruciate ligament rupture (CCLR) in bitches. BCS = body condition score; small = ≤10 kg; medium = 10–25 kg; large = ≥25 kg; early = <12 months old; late = ≥12 months old. Age is crossed out to indicate a non-significant association.

Timing relative to the first estrus was likewise associated with CCLR: females spayed before their first estrus had 4.51 times the odds of intact bitches (95% CI 3.14–6.49; *p* < 0.001) and 2.38 times the odds of those spayed after their first estrus (95% CI 1.61–3.50; *p* < 0.001). Bitches spayed after their first estrus also had increased odds compared with intact bitches (OR = 1.90, 95% CI 1.41–2.57; *p* < 0.001) (Figure 1).

#### 3.2.2. Hip Dysplasia

Body size and BCS were significantly associated with HD. Compared with bitches ≤10 kg, those weighing 10–25 kg and ≥25 kg had 2.09-fold (95% CI 1.22–3.55; *p* < 0.01) and 3.70-fold (95% CI 2.28–5.99; *p* < 0.001) higher odds of HD, respectively. In addition, bitches ≥25 kg had 1.77-fold higher odds than those weighing 10–25 kg (95% CI 1.25–2.52; *p* < 0.01). Each one-point increase in BCS increased the odds by 42% (OR = 1.42, 95% CI 1.26–1.59; *p* < 0.001). Age was not associated with HD.

Neutering was significantly associated with HD (OR = 2.27, 95% CI 1.66–3.11; *p* < 0.001). Early-spayed bitches had 3.78 times the odds of intact females (95% CI 2.42–5.89; *p* < 0.001) and 2.77 times the odds of late-spayed bitches (95% CI 1.62–4.72; *p* < 0.001). Late-spayed females did not differ significantly from intact females.

**Figure 2:**
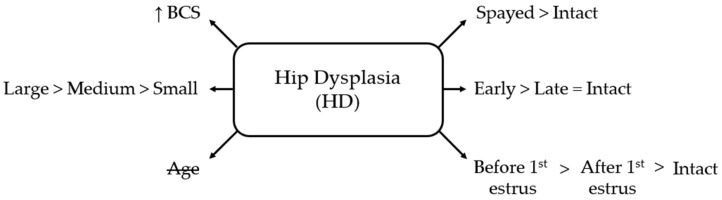
Schematic overview of factors associated with hip dysplasia (HD) in bitches. BCS = body condition score; small = ≤10 kg; medium = 10–25 kg; large = ≥25 kg; early = <12 months old; late = ≥12 months old. Age is crossed out to indicate a non-significant association.

Spaying before the first estrus doubled the odds of HD compared with post-estrus spaying (OR = 2.14, 95% CI 1.23–3.72; *p* < 0.01) and increased the odds more than threefold compared with intact females (OR = 3.50, 95% CI 2.11–5.82; *p* < 0.001). Bitches spayed after their first also had increased odds compared with intact bitches (OR = 1.64, 95% CI 1.04–2.56; *p* < 0.05) (Figure 2).

#### 3.2.3. Elbow Dysplasia

Body size and BCS were significantly associated with ED. Bitches ≥25 kg had 12.49 times the odds of ED compared with those ≤10 kg (95% CI 6.30–24.76; *p* < 0.001), and 2.85 times compared with those weighing 10–25 kg (95% CI 2.00–4.04; *p* < 0.001). Bitches between 10–25 kg also showed significantly increased odds compared with bitches ≤10 kg (OR = 4.39, 95% CI 2.11–9.12; *p* < 0.05). Each one-point increase in BCS increased the odds by 40% (OR = 1.40, 95% CI 1.25–1.56; *p* < 0.001). Age was not associated with ED.

Neutering was significantly associated with ED (OR = 2.38, 95% CI 1.77–3.19; *p* < 0.001). Early-spayed bitches had 4.43 times the odds of intact females (95% CI 2.86–6.86; *p* < 0.001) and 2.28 times the odds of late-spayed females (95% CI 1.39–3.74; *p* = 0.001). Late-spayed females also showed significantly increased odds compared with intact females (OR = 1.94, 95% CI 1.24–3.04; *p* < 0.01).

**Figure 3:**
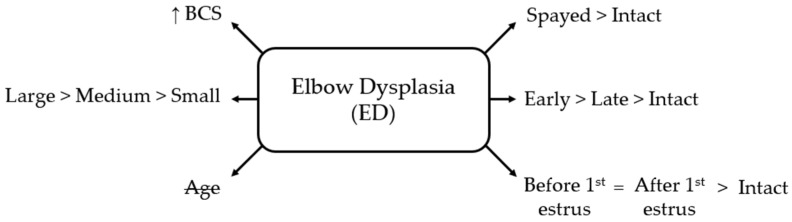
Schematic overview of factors associated with elbow dysplasia (ED) in bitches. BCS = body condition score; small = ≤10 kg; medium = 10–25 kg; large = ≥25 kg; early = <12 months old; late = ≥12 months old. Age is crossed out to indicate a non-significant association.

There was no significant difference between pre- and post-estrus spaying. However, both groups had higher odds than intact females (OR = 3.44, 95% CI 2.05–5.75; *p* < 0.001, and OR = 2.76, 95% CI 1.84–4.14; *p* < 0.001, respectively) (Figure 3).

### 3.3. Neoplastic Diseases

#### 3.3.1. Lymphoma

Body size and age were significantly associated with LSA. Bitches ≥25 kg had 2.71 times the odds of LSA compared with those ≤10 kg (95% CI 1.98–3.71; *p* < 0.01), and 1.82 times compared with those between 10–25 kg (95% CI 1.24–2.67; *p* < 0.01). There was no significant difference between bitches weighing 10–25 kg and bitches ≤10 kg. Increasing age raised the odds (OR = 1.13 per year, 95% CI 1.08–1.18; *p* < 0.001), while BCS showed no association.

Neutering significantly increased the odds of LSA (OR = 2.71, 95% CI 1.98–3.71; *p* < 0.001). Early-spayed bitches had 3.47 times the odds of intact females (95% CI 2.22–5.43; *p* < 0.001) and 1.68 times the odds of late-spayed females (95% CI 1.04–2.72; *p* < 0.05). Late-spayed females also had significantly higher odds than intact females (OR = 2.06, 95% CI 1.36–3.14; *p* = 0.001).

**Figure 4:**
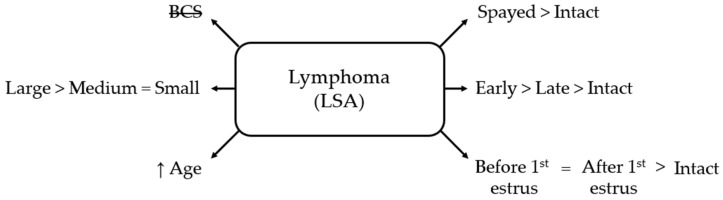
Schematic overview of factors associated with lymphoma (LSA) in bitches. BCS = body condition score; small = ≤10 kg; medium = 10–25 kg; large = ≥25 kg; early = <12 months old; late = ≥12 months old. BCS is crossed out to indicate a non-significant association.

Both pre- and post-estrus spaying increased LSA odds compared to intact females (OR = 2.86, 95% CI 1.68–4.87, and OR = 2.46, 95% CI 1.66–3.64, respectively, *p* < 0.001), with no significant difference between timing categories (Figure 4).

#### 3.3.2. Mast Cell Tumor

Body size was not significantly associated with MCT. In contrast, higher BCS (OR = 1.39, 95% CI 1.23–1.57; *p* < 0.001) and increasing age (OR = 1.21 per year, 95% CI 1.16–1.27; *p* < 0.001) were both associated with elevated odds.

Neutering was significantly associated with MCT (OR = 3.15, 95% CI 2.19–4.53; *p* < 0.001). Early-spayed females had three times the odds of intact bitches (OR = 3.06, 95% CI 1.83–5.10; *p* < 0.001) and twice the odds of late-spayed females (OR = 2.08, 95% CI 1.21–3.55; *p* < 0.01). Late-spayed females did not differ significantly from intact females (OR = 1.47, 95% CI 0.89–2.42; *p* > 0.05).

**Figure 5:**
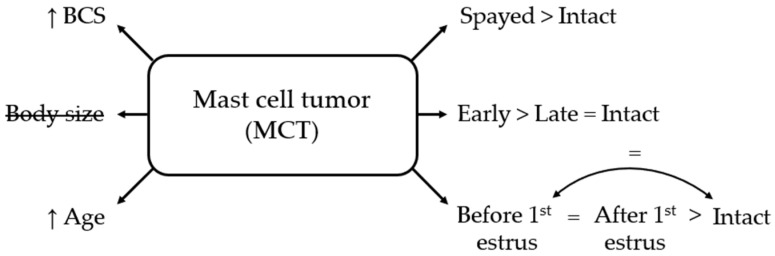
Schematic overview of factors associated with mast cell tumor (MCT) in bitches. BCS = body condition score; small = ≤10 kg; medium = 10–25 kg; large = ≥25 kg; early = <12 months old; late = ≥12 months old. Body size is crossed out to indicate a non-significant association.

There was no significant difference between pre- and post-estrus spaying, and between pre-estrus spaying and intact bitches. However, post-estrus spaying had increased odds compared to intact females (OR = 2.24, 95% CI 1.43–3.52; *p* < 0.001) (Figure 5).

#### 3.3.3. Hemangiosarcoma

Hemangiosarcoma risk was associated with body size and age. Bitches ≥25 kg were significantly more likely to develop HSA than those ≤10 kg (OR = 2.76, 95% CI 1.45–5.23; *p* < 0.01), whereas no significant differences were observed for the other body size categories. Age was associated with increased risk (OR = 1.33 per year, 95% CI 1.24–1.43; *p* < 0.001), whereas BCS showed no association.

Neutering increased the odds of HSA: spayed females had higher odds compared with intact females (OR = 4.77, 95% CI 2.81–8.08; *p* < 0.001). Early-spayed females had 5.82 times the odds of intact bitches (95% CI 2.84–11.92; *p* < 0.001) and 2.48 times the odds of late-spayed females (95% CI 1.26–4.85; *p* < 0.01). Late-spayed females also had significantly higher odds than intact females (OR = 2.35, 95% CI 1.21–4.56; *p* < 0.05).

**Figure 6:**
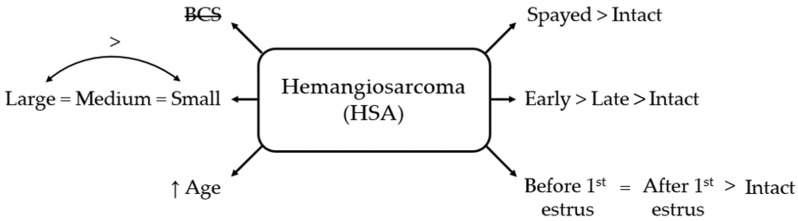
Schematic overview of factors associated with hemangiosarcoma (HSA) in bitches. BCS = body condition score; small = ≤10 kg; medium = 10–25 kg; large = ≥25 kg; early = <12 months old; late = ≥12 months old. BCS is crossed out to indicate a non-significant association.

Both pre- and post-estrus spaying increased HSA odds compared to intact females (OR = 3.95, 95% CI 1.66–9.41; *p* < 0.01, and OR = 3.19, 95% CI 1.71–5.96; *p* < 0.001), with no difference between timing categories (Figure 6).

#### 3.3.4. Osteosarcoma

Most (78.9%) osteosarcoma cases occurred in large bitches (≥25 kg). Body size and age (OR = 1.18 per year, 95% CI 1.10–1.27; *p* < 0.001) were significantly associated with OSA, whereas BCS showed no association. Bitches ≥25 kg had nearly 7 times the odds of OSA compared with those ≤10 kg (OR = 6.77, 95% CI 2.41–19.00; *p* < 0.001), and nearly 4 times the odds compared with those 10–25 kg (OR = 3.94, 95% CI 1.83–8.46; *p* < 0.001). No significant difference was observed between bitches weighing 10–25 kg and those weighing ≤10 kg.

Neutering was associated with higher odds of OSA (OR = 1.74, 95% CI 1.02–2.99; *p* < 0.05). Early-spayed bitches had 2.52 times the odds of intact females (95% CI 1.11–5.72; *p* < 0.05) and 3.38 times the odds of late-spayed females (95% CI 1.28–8.94; *p* < 0.05). Late-spayed females did not differ significantly from intact females.

**Figure 7:**
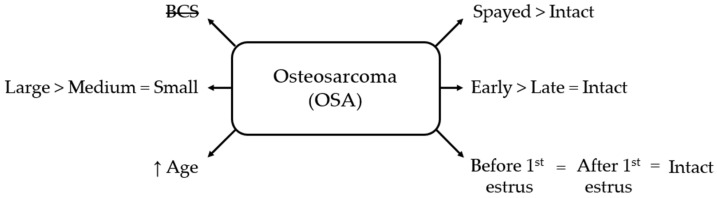
Schematic overview of factors associated with osteosarcoma (OSA) in bitches. BCS = body condition score; small = ≤10 kg; medium = 10–25 kg; large = ≥25 kg; early = <12 months old; late = ≥12 months old. BCS is crossed out to indicate a non-significant association.

Timing relative to the first estrus did not significantly influence OSA risk (Figure 7).

## 4. Discussion

The present study provides a comprehensive evaluation of how the timing of spaying relates to the risk of developing several orthopedic and neoplastic conditions in bitches. Based on a large cohort and multivariable models adjusting for key confounders, the results showed that spaying before 12 months of age was associated with increased odds across all seven investigated conditions. These findings reinforce the growing evidence that the peri-pubertal period constitutes a critical window of developmental sensitivity during which endocrine disruption may exert lasting effects on health [4,14,15,16,18,26]. This interpretation also aligns with the concept of developmental vulnerability described in the DOHaD concept, in which tissue maturation, hormonal regulation, immune development, and biomechanical adaptation converge during puberty [19,20]. Disruption of this coordinated physiology may not only influence orthopedic maturation but also long-term susceptibility to neoplastic disease.

This developmental sensitivity makes the mechanisms underlying orthopedic disorders particularly important to consider. Hip dysplasia and elbow dysplasia result from multifactorial interactions between genetic, developmental, and environmental factors that extend well beyond hormonal influences alone. Their expression depends on the interplay of genetic predisposition, skeletal conformation, body weight, growth rate, nutrition, exercise, and mechanical loading during early life [17,27,28]. Reproductive hormones therefore act more as modulators than as primary determinants. In our study, early spaying was associated with substantially higher odds of CCLR, HD, and ED even after adjusting for body weight, BCS, and age. These findings support the hypothesis that endocrine disruption during skeletal growth may exacerbate underlying predispositions. Reproductive hormones regulate growth plate closure, muscle development, ligament stiffness, and the coordination of bone and soft tissue maturation [29,30]. When removed before skeletal maturity, physeal closure is delayed, long bones continue to lengthen, joint angles may be altered, and ligamentous support becomes more lax [31]. These anatomical and biomechanical changes, though often subtle, may shift a dog from subclinical predisposition to overt clinical disease.

Because these developmental processes progress at different speeds across breeds, it is important to consider whether skeletal maturation aligns with the age at which spaying is recommended [32]. In many breeds, particularly large and giant ones, skeletal maturity occurs well after the first estrus and beyond 12 months of age, meaning that these developmental landmarks are not reliable indicators of musculoskeletal development [33]. As a result, a bitch may still be undergoing rapid growth at the time of gonadectomy, with open growth plates and incomplete maturation of joint-supporting structures. In our study, later gonadectomy (≥12 months) was associated with lower odds of orthopedic conditions than early gonadectomy, and in separate models, post-estrus gonadectomy was also associated with lower odds for some orthopedic outcomes compared with pre-estrus gonadectomy. However, even with delayed spaying, the odds remained higher than in intact females, suggesting that later timing was associated with only partial attenuation of the increased odds.

In addition to developmental timing, mechanical factors also contribute substantially to orthopedic outcomes. Body size and higher BCS were significantly associated with increased odds of CCLR, ED, and HD, consistent with the biomechanical strain imposed by (increased) body weight [22,31]. However, the association between early spaying and orthopedic disease remained strong even after adjusting for both body size and BCS, indicating that hormonal timing may exert an effect independent of mechanical factors. Because spaying is also known to reduce metabolic rate and increase the likelihood of weight gain [32,33], these mechanisms likely interact in clinically meaningful ways.

For neoplastic outcomes, early spaying was associated with increased odds of all investigated cancers. Spaying after 12 months was still associated with higher odds of LSA and HSA compared with intact females, whereas the odds of MCT and OSA did not differ significantly from those in intact females. These findings may suggest that longer lifetime exposure to endogenous ovarian hormones is associated with lower odds of certain malignancies, and that early hormonal withdrawal, rather than gonadectomy per se, may influence biological pathways involved in tumor development, such as immune regulation, angiogenesis, or proliferative signaling [34,35,36]. Recent canine studies further support the biological plausibility of such associations by reporting hormone-related receptor expression and proliferative responses in certain tumor types, although these mechanisms remain incompletely understood and appear to be tumor-specific [37,38,39]. Importantly, the number of cases for MCT (*n* = 165), HSA (*n* = 93), and OSA (*n* = 58) was relatively low, so these findings, particularly for HSA and OSA, should be interpreted with caution and confirmed in larger cohorts.

Given the developmental and multifactorial influences described above, the present findings suggest that neither a single chronological threshold nor a reproductive milestone fully captures biological development when considering the timing of gonadectomy. Associations with later gonadectomy timing were outcome-dependent and did not consistently approximate those observed in intact females.

An important limitation of this retrospective medical record-based study is the potential for outcome misclassification, particularly in the control group. Controls were defined as bitches without a recorded diagnosis of the investigated conditions during the study period. Because follow-up was not standardized and some dogs were seen only transiently, some control animals may have developed one of the investigated conditions later or may have been diagnosed at another practice. Similarly, prior or concurrent diagnoses may have been underreported in the anamnesis if they were unrelated to the reason for presentation. Such incomplete capture of historical disease could have resulted in under-ascertainment of cases and contamination of the control group. In addition, because case retrieval was intentionally restricted to predefined orthopedic and neoplastic conditions, the search strategy was not designed to capture the full spectrum of diagnoses in the medical records, and the findings should therefore not be generalized to orthopedic and neoplastic disease as a whole. Although BCS was confirmed by the veterinarian responsible for each case, formal inter-rater reliability could not be assessed, and some degree of variation in scoring between clinicians cannot be excluded. Because BCS was recorded at the time of clinical presentation rather than before disease onset, it should be interpreted as a concurrent clinical variable rather than a pre-existing causal risk factor. Potentially relevant environmental and management-related factors, such as diet, exercise, and housing conditions, were not consistently available in the medical records and could therefore not be included in the analyses. The age variable combined age at symptom onset with age at diagnosis when symptom onset was not documented. Although this approach was intended to better approximate disease emergence in a referral population, it introduced heterogeneity and should therefore be interpreted with caution. Finally, because the study population was derived from a single veterinary teaching hospital, it may not fully represent the geographic distribution or referral patterns of the wider Belgian dog population. These limitations are inherent to retrospective analyses and highlight the need for prospective studies with standardized follow-up, more complete reproductive histories, and collection of environmental and management-related variables. Moreover, the present study was not designed to model gonadectomy as a time-varying exposure or to determine whether the interval between gonadectomy and disease onset influenced risk, as these questions would require a longitudinal time-to-event approach with standardized follow-up. Age at gonadectomy and timing relative to the first estrus were analyzed separately because they reflect related but distinct aspects of development and were considered complementary rather than interchangeable indicators. Accordingly, potential interactions between these variables were not assessed. The 12-month threshold was used as a standardized chronological cut-off for a heterogeneous multi-breed population, while timing relative to the first estrus provided an additional marker of reproductive maturation. However, neither variable should be interpreted as a precise proxy for biological maturity, particularly in large and giant breeds, in which pubertal and skeletal development are influenced by body size [40,41]. In addition, all breeds were analyzed as a pooled population, precluding assessment of breed-specific predispositions [42,43,44]. Although weight class was included to account for differences in body size, this approach cannot fully distinguish the effects of body size from breed-related genetic susceptibility. Accordingly, residual confounding related to breed may have influenced the observed associations. Given the high breed diversity of the study population and the limited number of individuals in many breeds, a more detailed breed-specific analysis was not feasible. Consequently, while the observed associations reflect population-level effects, the magnitude of risk associated with gonadectomy timing may differ substantially at the individual breed level.

Finally, these findings should not be interpreted as an argument against spaying. The health benefits of gonadectomy in bitches remain well established and clinically meaningful. Rather, the present study supports consideration of a more individualized, context-specific approach to decision-making in which the timing of surgery is adapted to the dog’s body size, individual risk factors, and intended reproductive use. Encouraging individualized decision-making regarding the timing of spaying, rather than routine early gonadectomy, may help balance long-term orthopedic and neoplastic outcomes while preserving the recognized medical and population-control benefits of the procedure.

## Figures and Tables

**Table 1:** Descriptive characteristics of the study population by outcome group and neuter status.

Condition	Neuter Status	*n*	Body Weight Category			BCS	Age
			≤10 kg	10–25 kg	≥25 kg		
CCLR	Intact	181	34	22	125	5	57.1
			(18.8%)	(12.1%)	(69.1%)	(3–6)	(27.0–90.0)
	Spayed	387	65	60	262	5	72.5
			(16.8%)	(15.5%)	(67.7%)	(4–6)	(44.0–99.5)
HD	Intact	69	12	20	37	5	64.1
			(17.4%)	(28.9%)	(53.6%)	(4–6)	(32.0–90.6)
	Spayed	137	9	31	92	5	62.2
			(6.8%)	(23.5%)	(69.7%)	(4–6)	(37.3–94.9)
ED	Intact	79	7	16	56	5	51.0
			(8.9%)	(20.2%)	(70.9%)	(3–6)	(26.0–80.6)
	Spayed	164	2	30	131	5	50.2
			(1.2%)	(18.4%)	(80.4%)	(5–7)	(33.0–83.0)
LSA	Intact	65	11	16	38	5	78.0
			(16.9%)	(24.6%)	(58.5%)	(3–6)	(54.6–110.3)
	Spayed	154	30	37	86	5	92.0
			(19.6%)	(24.2%)	(56.2%)	(4–5)	(67.5–118.5)
MCT	Intact	44	11	9	24	5	97.0
			(25.0%)	(20.5%)	(54.5%)	(3–7)	(70.3–131.6)
	Spayed	121	32	30	59	5	111.1
			(26.4%)	(24.8%)	(48.8%)	(4–7)	(83.5–133.3)
HSA	Intact	18	2	5	11	5	116.0
			(11.1%)	(27.8%)	(61.1%)	(4–6)	(94.9–130.3)
	Spayed	75	10	19	44	5	125.0
			(13.7%)	(26.0%)	(60.3%)	(4–6)	(100.0–145.9)
OSA	Intact	23	2	4	17	4	99.3
			(8.7%)	(17.4%)	(73.9%)	(3–5)	(23.0–123.0)
	Spayed	35	2	4	28	5	113.0
			(5.9%)	(11.8%)	(82.3%)	(4–6)	(85.0–144.0)
Control	Intact	585	141	160	281	5	50.0
			(24.2%)	(27.5%)	(48.3%)	(4–5)	(22.2–77.0)
	Spayed	511	144	172	193	5	86.9
			(28.3%)	(33.8%)	(37.9%)	(4–5)	(47.1–120.6)

Abbreviations: CCLR = cranial cruciate ligament rupture; HD = hip dysplasia; ED = elbow dysplasia; LSA = lymphoma; MCT = mast cell tumor; HSA = hemangiosarcoma; OSA = osteosarcoma; BCS = body condition score. Categorical variables are shown as *n* (%), with percentages calculated within each condition and neuter-status subgroup. BCS and age are presented as median (interquartile range). Age is expressed in months.

## Data Availability

The data presented in this study are available on request from the corresponding author.

## Abbreviations

The following abbreviations are used in this manuscript:

DOHaD	Developmental Origins of Health and Disease
CCLR	Cranial Cruciate Ligament Rupture
HD	Hip Dysplasia
ED	Elbow Dysplasia
LSA	Lymphoma
MCT	Mast Cell Tumor
HSA	Hemangiosarcoma
OSA	Osteosarcoma
BCS	Body Condition Score
IQR	Interquartile range
OR	Odds Ratio

## References

[1] Diesel G., Brodbelt D., Laurence C. (2010). Survey of veterinary practice policies and opinions on neutering dogs. Vet. Rec..

[2] Arlt S., Wehrend A., Reichler I.M. (2017). Neutering of female dogs-old and new insights into Pros and Cons. Tierarztl. Prax. Ausg. K Kleintiere Heimtiere.

[3] Downes M.J., Devitt C., Downes M.T., More S.J. (2015). Neutering of cats and dogs in Ireland; pet owner self-reported perceptions of enabling and disabling factors in the decision to neuter. Peerj.

[4] Kustritz M.V.R. (2007). Determining the optimal age for gonadectomy of dogs and cats. Javma-J. Am. Vet. Med. A.

[5] Fossati P. (2024). Spay/neuter laws as a debated approach to stabilizing the populations of dogs and cats: An overview of the European legal framework and remarks. J. Appl. Anim. Welf. Sci..

[6] Egenvall A., Hedhammar A., Bonnett B.N., Olson P. (1999). Survey of the Swedish dog population: Age, gender, breed, location and enrollment in animal insurance. Acta Vet. Scand..

[7] Howe L.M. (2015). Current perspectives on the optimal age to spay/castrate dogs and cats. Vet. Med..

[8] Trevejo R., Yang M., Lund E.M. (2011). Epidemiology of surgical castration of dogs and cats in the United States. J. Am. Vet. Med. Assoc..

[9] Hart B.L., Hart L.A., Thigpen A.P., Willits N.H. (2014). Long-term health effects of neutering dogs: Comparison of Labrador Retrievers with Golden Retrievers. PLoS ONE.

[10] Yates D., Leedham R. (2019). Prepubertal neutering in cats and dogs. Pract..

[11] Houlihan K.E. (2017). A literature review on the welfare implications of gonadectomy of dogs. J. Am. Vet. Med. Assoc..

[12] Romagnoli S., Krekeler N., de Cramer K., Kutzler M., McCarthy R., Schaefer-Somi S. (2024). WSAVA guidelines for the control of reproduction in dogs and cats. J. Small Anim. Pract..

[13] Reichler I.M., Welle M., Eckrich C., Sattler U., Barth A., Hubler M., Nett-Mettler C.S., Jochle W., Arnold S. (2008). Spaying-induced coat changes: The role of gonadotropins, GnRH and GnRH treatment on the hair cycle of female dogs. Vet. Dermatol..

[14] Urfer S.R., Kaeberlein M. (2019). Desexing Dogs: A Review of the Current Literature. Animals.

[15] Torres de la Riva G., Hart B.L., Farver T.B., Oberbauer A.M., Messam L.L., Willits N., Hart L.A. (2013). Neutering dogs: Effects on joint disorders and cancers in golden retrievers. PLoS ONE.

[16] Robinson K.L., Bryan M.E., Atkinson E.S., Keeler M.R., Hahn A.W., Bryan J.N. (2020). Neutering is associated with developing hemangiosarcoma in dogs in the Veterinary Medical Database: An age and time-period matched case-control study (1964–2003). Can. Vet. J..

[17] Smith G.K., Mayhew P.D., Kapatkin A.S., McKelvie P.J., Shofer F.S., Gregor T.P. (2001). Evaluation of risk factors for degenerative joint disease associated with hip dysplasia in German Shepherd Dogs, Golden Retrievers, Labrador Retrievers, and Rottweilers. J. Am. Vet. Med. Assoc..

[18] Belanger J.M., Bellumori T.P., Bannasch D.L., Famula T.R., Oberbauer A.M. (2017). Correlation of neuter status and expression of heritable disorders. Canine Genet. Epidemiol..

[19] Gaillard V., Chastant S., England G., Forman O., German A.J., Suchodolski J.S., Villaverde C., Chavatte-Palmer P., Peron F. (2022). Environmental risk factors in puppies and kittens for developing chronic disorders in adulthood: A call for research on developmental programming. Front. Vet. Sci..

[20] Hoffman D.J., Reynolds R.M., Hardy D.B. (2017). Developmental origins of health and disease: Current knowledge and potential mechanisms. Nutr. Rev..

[21] Zink M.C., Farhoody P., Elser S.E., Ruffini L.D., Gibbons T.A., Rieger R.H. (2014). Evaluation of the risk and age of onset of cancer and behavioral disorders in gonadectomized Vizslas. J. Am. Vet. Med. Assoc..

[22] Hart B.L., Hart L.A., Thigpen A.P., Willits N.H. (2020). Assisting Decision-Making on Age of Neutering for 35 Breeds of Dogs: Associated Joint Disorders, Cancers, and Urinary Incontinence. Front. Vet. Sci..

[23] Salt C., Morris P.J., German A.J., Wilson D., Lund E.M., Cole T.J., Butterwick R.F. (2017). Growth standard charts for monitoring bodyweight in dogs of different sizes. PLoS ONE.

[24] Freeman L., Becvarova I., Cave N., MacKay C., Nguyen P., Rama B., Takashima G., Tiffin R., van Beukelen P., Yathiraj S. (2011). WSAVA Nutritional Assessment Guidelines. J. Feline Med. Surg..

[25] Chun J.L., Bang H.T., Ji S.Y., Jeong J.Y., Kim M., Kim B., Lee S.D., Lee Y.K., Reddy K.E., Kim K.H. (2019). A simple method to evaluate body condition score to maintain the optimal body weight in dogs. J. Anim. Sci. Technol..

[26] Smith A.N. (2014). The role of neutering in cancer development. Vet. Clin. Small Anim. Pract..

[27] Ginja M.M., Silvestre A.M., Gonzalo-Orden J.M., Ferreira A.J. (2010). Diagnosis, genetic control and preventive management of canine hip dysplasia: A review. Vet. J..

[28] Schachner E.R., Lopez M.J. (2015). Diagnosis, prevention, and management of canine hip dysplasia: A review. Vet. Med..

[29] Mills E.G., Yang L., Nielsen M.F., Kassem M., Dhillo W.S., Comninos A.N. (2021). The Relationship Between Bone and Reproductive Hormones Beyond Estrogens and Androgens. Endocr. Rev..

[30] Nilsson O., Weise M., Landman E.B., Meyers J.L., Barnes K.M., Baron J. (2014). Evidence that estrogen hastens epiphyseal fusion and cessation of longitudinal bone growth by irreversibly depleting the number of resting zone progenitor cells in female rabbits. Endocrinology.

[31] Salmeri K.R., Bloomberg M.S., Scruggs S.L., Shille V. (1991). Gonadectomy in immature dogs: Effects on skeletal, physical, and behavioral development. J. Am. Vet. Med. Assoc..

[32] Kumar K., Mogha I.V., Aithal H.P., Amarpal, Kinjavdekar P., Singh G.R., Pawde A.M., Setia H.C. (2009). Determinants of bone mass, density and growth in growing dogs with normal and osteopenic bones. Vet. Res. Commun..

[33] Tryfonidou M.A., Hazewinkel H.A., Riemers F.M., Brinkhof B., Penning L.C., Karperien M. (2010). Intraspecies disparity in growth rate is associated with differences in expression of local growth plate regulators. Am. J. Physiol. Endocrinol. Metab..

[34] Fields M.J., Shemesh M. (2004). Extragonadal luteinizing hormone receptors in the reproductive tract of domestic animals. Biol. Reprod..

[35] Beijerink N.J., Buijtels J.J., Okkens A.C., Kooistra H.S., Dieleman S.J. (2007). Basal and GnRH-induced secretion of FSH and LH in anestrous versus ovariectomized bitches. Theriogenology.

[36] Kutzler M.A. (2023). Understanding the effects of sustained supraphysiologic concentrations of luteinizing hormone in gonadectomized dogs: What we know and what we still need to learn. Theriogenology.

[37] Dilley K.N., Wong A., Kent M.S., Steffey M.A., Yellowley C.E. (2022). Expression of Sex Hormone Receptors in Canine Osteosarcoma. Vet. Sci..

[38] Weinman M.A., Fischer J.A., Jacobs D.C., Goodall C.P., Bracha S., Chappell P.E. (2019). Autocrine production of reproductive axis neuropeptides affects proliferation of canine osteosarcoma in vitro. Bmc Cancer.

[39] Bugiel-Stabla K., Agnoli C., Pawlak A. (2024). Estrogen receptors alpha and beta expression in different canine cancer types with an emphasis on hematopoietic malignancies. Vet. Res. Commun..

[40] Roccaro M., Diana A., Linta N., Rinnovati R., Freo M., Peli A. (2021). Limb development in skeletally-immature large-sized dogs: A radiographic study. PLoS ONE.

[41] Hawthorne A.J., Booles D., Nugent P.A., Gettinby G., Wilkinson J. (2004). Body-weight changes during growth in puppies of different breeds. J. Nutr..

[42] Egenvall A., Nodtvedt A., von Euler H. (2007). Bone tumors in a population of 400 000 insured Swedish dogs up to 10 y of age: Incidence and survival. Can. J. Vet. Res..

[43] Comazzi S., Marelli S., Cozzi M., Rizzi R., Finotello R., Henriques J., Pastor J., Ponce F., Rohrer-Bley C., Rutgen B.C. (2018). Breed-associated risks for developing canine lymphoma differ among countries: An European canine lymphoma network study. BMC Vet. Res..

[44] Adams P., Bolus R., Middleton S., Moores A.P., Grierson J. (2011). Influence of signalment on developing cranial cruciate rupture in dogs in the UK. J. Small Anim. Pract..

